# Botulinum Toxin A: A Review of Potential Uses in Treatment of Female Urogenital and Pelvic Floor Disorders

**DOI:** 10.31486/toj.19.0076

**Published:** 2020

**Authors:** Laurephile Desrosiers, Leise R. Knoepp

**Affiliations:** ^1^ Department of Obstetrics and Gynecology, Division of Female Pelvic Medicine and Reconstructive Surgery, Ochsner Clinic Foundation, New Orleans, LA; ^2^ The University of Queensland Faculty of Medicine, Ochsner Clinical School, New Orleans, LA

**Keywords:** *Botulinum toxins–type A*, *pelvic floor disorders*, *pelvic pain*, *urogenital system*

## Abstract

**Background:** Botulinum toxin is an injectable neuromodulator that inhibits transmission between peripheral nerve endings and muscle fibers, resulting in muscle paralysis. Botulinum toxin type A is the most common form of botulinum toxin used in clinical practice.

**Methods:** In this review, we examine the mechanism of action, formulations, common clinical use in the genital-urinary tract, and potential clinical use in pelvic floor disorders of botulinum toxin type A.

**Results:** Several aspects of botulinum toxin A make it a favorable therapeutic tool, including its accessibility, its longevity, and its impermanence and reversibility of resultant chemodenervation in a relatively short and safe manner. Although botulinum toxin A has well-established efficacy in treating refractory overactive bladder and neurogenic detrusor overactivity, its use in pelvic floor disorders is still in its infancy.

**Conclusion:** The efficacy of botulinum toxin A for treating pelvic pain, voiding dysfunction, muscle pain and dysfunction, and certain colorectal-related pain issues shows promise but requires additional rigorous evaluation.

## INTRODUCTION

Botulinum toxin is an injectable neuromodulator derived from neurotoxins produced by *Clostridium botulinum*, a gram-positive, rod-shaped anaerobic bacterium responsible for the production of the botulism-inducing neurotoxin. This neurotoxin inhibits transmission between peripheral nerve endings and muscle fibers, resulting in weakness or flaccid paralysis of skeletal muscle. The clinical potential of *C botulinum* was recognized initially in 1897 when the toxin produced by the organism was identified as the etiologic agent of botulism.^1,2^ Since then, 7 serotypes of botulinum toxin (A, B, C1, D, E, F, and G), produced by different strains of the bacterium, have been identified. Although the pharmacologic properties of the serotypes differ, suggesting differences in clinical efficacy, only serotypes A and B are available for clinical use. Several aspects of botulinum toxin A make it a favorable therapeutic tool, such as injection longevity (effects last 3 to 4 months when injected into skeletal muscle and 6 to 9 months when injected into smooth muscle) and impermanence and reversibility (recovery of resultant chemodenervation after 3 to 6 months because of synaptic turnover).^3,4^ Botulinum toxin type A is the most common form of botulinum toxin used in clinical practice^5-7^ and is the primary focus of this review in which we examine its mechanism of action, formulations, common clinical use in the genital-urinary tract, and potential clinical use in pelvic floor disorders.

## MECHANISM OF ACTION

**Figure 1.**
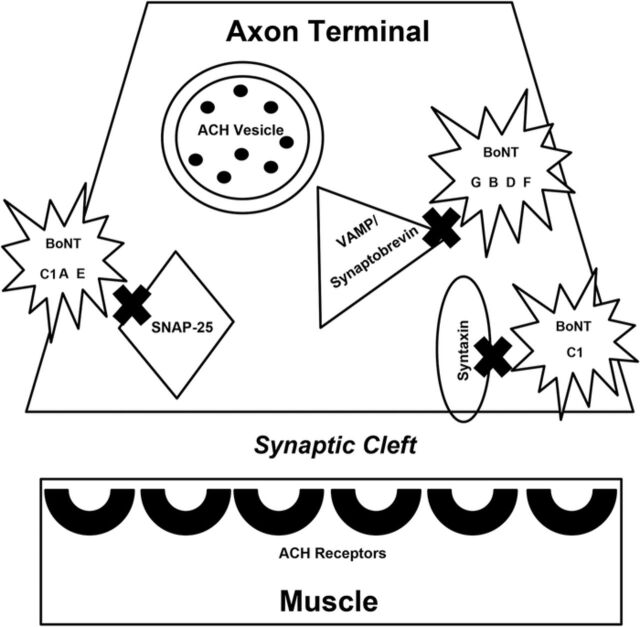
**Mechanism of action of different botulinum toxin (BoNT) types. The acetylcholine (ACH) vesicle binds the soluble N-ethylmaleimide-sensitive factor attachment protein receptor (SNARE) complex (synaptosome-associated protein of 25kDa [SNAP-25]), vesicle-associated membrane protein [VAMP]/synaptobrevin, syntaxin) to allow exocytosis of the neurotransmitter ACH from the axon into the synaptic cleft where it can bind end receptors on the muscle, allowing muscle contraction. Different types of BoNT affect different parts of the SNARE complex, preventing exocytosis of ACH, resulting in muscle paralysis. This diagram shows the specific sites affected by each BoNT type.**

The clinical utility of botulinum toxin stems from its ability to prevent muscular contraction through inhibiting the release of acetylcholine from peripheral nerve cells into their neuromuscular junctions. In short, release of acetylcholine is suppressed sequentially as follows: toxin receptor binding and internalization, followed by cleaving and inhibition of acetylcholine. Botulinum toxins are peptides composed of one heavy chain and one light chain. After the heavy chain of the injected toxin binds to the terminal ends of a neuron, the peptide enters the cytoplasm through endocytosis. Once within the cytoplasm, the light chain cleaves components of soluble N-ethylmaleimide-sensitive factor attachment protein receptor (SNARE), a complex of proteins necessary for the exocytosis of acetylcholine. The sites of cleavage within the SNARE protein complex differ among the serotypes; while botulinum toxin types A, C1, and E cleave synaptosome-associated protein of 25kDa (SNAP-25), serotypes B, D, F, and G cleave vesicle-associated membrane protein (VAMP), also known as synaptobrevin (Figure 1).^8^ As a result of cleavage, acetylcholine remains within the neuron where it is unable to bind to receptors on muscle fibers and stimulate muscle contraction (chemodenervation).

The inhibitory effect of botulinum toxin is temporary; recovery of muscular function often becomes clinically evident approximately 3 to 6 months after treatment as the neuromuscular junction begins to recover. The development of new collateral nerve endings may be partially responsible for this recovery. However, these terminal buds appear to be transient, and recovery of the original nerve terminal eventually occurs, resulting in reversal of the toxin's effect.^8-10^

## FORMULATIONS

As stated previously, 2 serotypes of botulinum toxin have been formulated for clinical use: type A (the most common serotype used for cosmetic applications) and type B. Commercially available formulations of botulinum toxin type A in North America include onabotulinumtoxinA, abobotulinumtoxinA, and incobotulinumtoxinA. RimabotulinumtoxinB is the established name for botulinum toxin type B.^11^ Of the formulations of botulinum toxin type A that are available in 2020 or in development, onabotulinumtoxinA has been studied most extensively.

## APPROVED USES FOR UROGENITAL DISORDERS

Botulinum toxin is US Food and Drug Administration (FDA)-indicated for multiple purposes, but with regard to urogenital and pelvic floor disorders (Table), in 2020, it is only approved for (1) treatment of overactive bladder with symptoms of urinary urge incontinence, urgency, and frequency in adults who have an inadequate response to or are intolerant of an anticholinergic medication, and (2) treatment of urinary incontinence because of detrusor overactivity associated with a neurologic condition (eg, spinal cord injury, multiple sclerosis) in adults who have an inadequate response to or are intolerant of an anticholinergic medication.^12^ Botox (onabotulinumtoxinA) has been FDA approved for treatment of neurogenic detrusor overactivity since 2011 and for refractory overactive bladder symptoms since 2013,^13^ increasing the likelihood of insurance coverage, and thus the affordability of and access to this treatment for pelvic floor indications. Additionally, studies have shown Botox to be cost effective relative to nonselective anticholinergics, advocating for consideration as a first-line treatment for overactive bladder.^14^

**Table.** Clinical Applications of Botox in the Treatment of Urogenital and Pelvic Floor Disorders

Disorder	Treatment, IU	Location	Level of Recommendation
Urologic			
Idiopathic detrusor overactivity	100	Bladder	A
Neurogenic detrusor overactivity	200	Bladder	A
Dyssynergic sphincter deficiency	200	Bladder	C
Interstitial cystitis/painful bladder syndrome	100-200	Bladder	C
Gynecologic			
Pudendal neuralgia	15-60	Muscle	C
High tone pelvic floor dysfunction	20-100	Muscle	C
Vestibulodynia	20-100	Perineum	B
Colorectal			
Anal fissure	20-300	Internal anal sphincter	B
Puborectalis syndrome (anismus)	12-100	3 and 9 o’clock by puborectalis muscle	B
Hemorrhoids and healing	20-100	Internal anal sphincter	B
Anal pain	20-200	Muscle and/or internal anal sphincter	B

Note: Levels of recommendation are (A) based on at least one randomized clinical trial as part of the scientific evidence and overall represents good and consistent scientific evidence; (B) methodologically correct, but nonrandomized clinical trials on the topic overall are based on limited and inconsistent scientific evidence; and (C) consensus and expert opinions or committee recommendations, but high-quality directly applicable clinical studies are lacking.

### Overactive Bladder

Botox has been effective in treating refractory overactive bladder and is usually given at doses of 100 to 300 IU^15-17^ every 6 to 12 months, with most clinicians administering 100-IU doses to minimize side effects.^4^ Compelling evidence from a 2011 meta-analysis noted significant reduction in urinary frequency and urge incontinence at 6 and 12 weeks compared with placebo treatment.^18^ Multiple studies have also shown that this treatment regimen has some duration, although time of efficacy after each injection has been variable.^19,20^ In initial trials, time to retreatment after injection of 100 IU of injected onabotulinumtoxinA was 19 to 24 weeks, leading the manufacturer to recommend passage of at least 12 weeks between injections.^12^ Predictors of treatment success have been assessed, although also with inconsistent results. Female participants (especially <65 years old) with low overactive bladder symptom scores on preoperative questionnaires, subjects with wet overactive bladder, and subjects who stopped other treatments before undergoing Botox injection have all been found to have higher success rates with treatment.^21-23^

### Neurogenic Detrusor Overactivity

Botox injections have also been useful in the treatment of neurogenic detrusor overactivity, while protecting the upper urinary tract and improving quality of life through decreasing overall urge incontinence episodes.^24^ In a large meta-analysis, subjects receiving treatment experienced a reduction in urinary urge incontinence episodes per week and maximum detrusor voiding pressure, while increasing maximum cystometric capacity.^25^ Although doses of 200 to 300 IU have been studied, injection of 200 IU has been found to have optimized efficacy while minimizing potential side effects.^26^

Injection of Botox for urinary symptoms is generally through a rigid or flexible cystoscope, with the total dose divided and administered in 10 to 20 small injections into the detrusor muscle of the bladder. The specifics about injection technique, such as the optimal intravesical anatomic location and the number of injections, are still topics of debate, although limited studies have not demonstrated increased rates of urinary retention, frequency of urinary tract infections, or vesicoureteral reflux after trigonal injection.^27,28^ A European consensus report includes some discussion about the efficacy of suburothelial vs intradetrusor injections.^29^

Although Botox treatment is typically considered safe for use in the treatment of overactive bladder and neurogenic detrusor overactivity, the potential for adverse effects exists. Most significantly, high injection doses (300 to 1,000 IU) have (rarely) been associated with generalized weakness^29^ and life-threatening toxicity, similar to typical botulism, resulting in issuance of an FDA warning in 2009.^13^ More commonly, Botox injections for urinary issues can result in increased rates of urinary retention and increased frequency of urinary tract infections.^12^

## POTENTIAL USES FOR PELVIC FLOOR DISORDERS

As of 2020, Botox is not FDA approved for the pelvic floor disorders discussed in this section; however, data show that the use of Botox in these areas is promising.

### Female Pelvic Pain

Pelvic pain in women, which is a major cause of morbidity and disability for patients and results in significant costs to health services, is a complex and challenging dilemma. When the cause of pain can be defined, the majority of women experiencing acute pain can be effectively treated with medical and surgical interventions. However, in a subset of patients, diagnosing the cause of pain can be evasive, and these patients often have decreased success and dissatisfaction with treatment. As a further complication, chronic pelvic pain is not only influenced by multiple systems (neurologic, musculoskeletal, endocrine), but a growing body of evidence also shows a strong behavioral and psychological component. Given its potential analgesic effects, Botox may be a potential treatment modality for pelvic pain. These analgesic effects are likely attributable to secondary effects achieved from muscle paralysis, which leads to improved blood flow, decompression of nerve fibers by abnormally contracting muscle, and downregulation of nociceptive neurons.

#### Pudendal Neuralgia

Sacral roots S2 to S4 coalesce to form the pudendal nerve. Typically, the pudendal nerve courses underneath the piriformis muscle and between the sacrospinous and sacrotuberous ligaments at the level of the ischial spine. The pudendal nerve then travels through the Alcock canal between the obturator internus and levator ani muscles and divides into 3 branches: the dorsal nerve of the clitoris (or penis), the perineal nerve, and the inferior rectal nerve. The 2 most common areas of nerve entrapment occur at the level of the ischial spine and the Alcock canal. Damage to the pudendal nerve can result in unilateral or bilateral pelvic pain in females, involving the vulva, vagina, clitoris, or bladder. Pudendal nerve dysfunction because of entrapment or compression has been postulated to not only lead to chronic pain but also to negatively impact the afferent, efferent, and autonomic signals between pelvic organs that are essential for maintaining their proper functions.^30^

#### Levator Myalgia

Levator myalgia is a condition characterized by burning pain or tenesmus of the perineum or rectum. In a 2004 study by Jarvis et al, 12 patients with a minimum 2-year history of chronic pelvic pain and pelvic floor hypertonicity underwent injection of 40 IU of botulinum toxin A into bilateral levator ani muscles.^30^ Visual analog scale (VAS) scores and muscle manometry showed improvement at 4 weeks, which was maintained at the 12-week assessment. Abbott et al conducted a double-blind randomized controlled trial in which women received pelvic floor injections of 80 IU of Botox (n=30) vs saline (n=30).^31^ Significant improvement in VAS scores and muscle manometry was noted in the Botox recipients. Adelowo et al published a retrospective review of 31 subjects who received Botox injections of 100 to 300 IU for myofascial pain.^32^ Primary outcomes were self-reported pain on muscle palpation and symptom improvement. The median pain noted with levator palpation was significantly lower at 6 weeks (*P*<0.0001) postinjection and remained significantly decreased at 12 weeks (*P*<0.0001). Within the cohort, 58% opted to have a repeat injection, with a median time to repeat injection of 4 months. Adverse side effects included de novo urinary retention (10.3%), fecal incontinence (6.9%), and constipation and/or rectal pain (10.3%), all of which resolved spontaneously. In 2013, a prospective study by Nesbitt-Hawes et al assessed successive botulinum toxin A injections in women with pelvic floor disorders.^33^ Thirty-seven patients received injections of 100 IU of botulinum toxin A into the puborectalis and pubococcygeus muscles, with 30% receiving 2 or more injections. The VAS scores and vaginal pressure measured by manometry were evaluated at 0, 4, 12, and 26 weeks. Both single and repeated injections demonstrated a statistically significant reduction in dyspareunia (*P*=0.001) as measured by VAS scores, but vaginal pressure improvement was only noted in the multiple injection group (*P*=0.02). No differences in dysmenorrhea or dyschezia rates were observed. In 2015, Morrissey et al selected subjects with chronic pelvic pain and high muscle tone pelvic floor dysfunction who had failed conventional therapy to receive Botox injections (maximum dose 300 IU) through electromyography guidance, allowing specific localization of spasticity in pelvic floor muscles.^34^ Posttreatment vaginal manometry demonstrated significant decreases in resting pressure and maximum contraction pressures (*P*<0.05). Adverse effects after injections included constipation (28%), stress urinary incontinence (4.8%), and fecal incontinence (4.8%).

#### Vulvodynia

Vulvodynia is characterized by burning pain in the vulvar area, persistent for at least 3 months, in the absence of other relevant dermatologic or neurologic findings. Vulvodynia negatively impacts quality of life, and various treatment modalities, including surgery and yttrium aluminum garnet (YAG) lasers, have been useful in reducing this type of pain.^35,36^ Postulated efficacy in this subset of patients with pelvic pain is based on the effect of Botox at the neuromuscular junction and within parasympathetic and sympathetic neural synapses. The first report of Botox used to treat vulvovaginal pain was in 1997, when Brin and Vapnek published their data on successful treatment of vaginismus with Botox.^37^ In 2000, after injection of Botox vs saline in a small population, Shafik and El-Sibai found superiority in pain control for the Botox group.^38^ In 2004, Gunter et al described successful management of refractory vulvodynia with a combination of surgery and Botox.^39^ Since that time, other studies have reported improvement in vulvodynia,^40-45^ while others have found no change in symptoms.^46^ In 2017, Halder et al reported improved VAS scores after treatment with combined Botox and physical therapist–mediated myofascial release.^47^ Posttreatment complications included constipation (8%), urinary retention (2%), and urinary tract infections (4%). In an effort to improve injection success, Nesbitt-Hawes et al developed a technique using 4-dimensional sonogram guidance to aid injection.^48^ Their findings have shown good feasibility while allowing accurate placement of the injection within the target muscle in women with pelvic floor muscle overactivity.

#### Interstitial Cystitis

Now more commonly termed bladder pain syndrome, interstitial cystitis is a type of chronic pain that affects the bladder and pelvic floor. Symptoms vary but often include persistent urinary urgency and frequency, dyspareunia, bladder base tenderness, and levator myalgia. Interstitial cystitis/bladder pain syndrome also has strong associations with depression and anxiety, irritable bowel syndrome, and fibromyalgia. Although the exact pathophysiology of interstitial cystitis/bladder pain syndrome is unknown, several theories exist; the most common faults disruption in the glycosaminoglycan of the basement membrane of the bladder that leads to greater infiltration of irritating substances. Generally, glomerulations, petechial hemorrhages, or other signs of inflammation can be seen on cystoscopy, but the identification of a Hunner ulceration has greater specificity for interstitial cystitis/bladder pain syndrome diagnosis and is usually associated with more severe symptoms. To date, 3 double-blind randomized controlled studies have evaluated the use of botulinum toxin A in the treatment of interstitial cystitis/bladder pain syndrome. Kuo and Kuo compared 100 IU Botox vs saline, and the botulinum toxin A group showed a significantly greater reduction in VAS scores and global improvement response assessment at 3 months.^49^ Chuang and Kuo evaluated the use of intravesical instillation of a liposomal lipotoxin form of Botox in patients with interstitial cystitis/bladder pain syndrome.^50^ Significant improvement was noted at 4 weeks; however, no differences in endpoints were appreciated among the groups at 12 weeks. In 2018, Pinto et al also found a significant reduction in VAS scores in the subjects receiving botulinum toxin A vs placebo.^51^ Studies by Rappaport et al and Jiang et al assessed hydrogel-based delivery systems and trigonal intravesical injections, respectively, and the studies demonstrated safety and at least short-term efficacy with both techniques.^52,53^ In 2020, no consensus exists regarding the efficacy of treating interstitial cystitis/bladder pain syndrome with botulinum toxin A. However, studies suggest a trend toward improvement in symptoms. Well-designed studies are needed to further evaluate this trend, as well as to define ideal dosage, injection location, and alternate delivery systems.

#### Postoperative Pelvic Pain

Postoperative pelvic pain is complex in its presentation and treatment. Studies concerning the utility of Botox injections in treating this type of pain are limited, but Park and Paraiso reported successfully treating a patient presenting with de novo refractory dyspareunia after pelvic floor reconstructive surgery.^45^ More research is needed to fully define the role of Botox in the treatment of postoperative pelvic pain.

As the discussion in this section has demonstrated, the indications for Botox treatment are not entirely clear for patients experiencing different types of pelvic pain. However, treatment of such pain with Botox shows promise in conditions such as chronic pelvic floor muscle spasm (primary or etiology) and interstitial cystitis/bladder pain syndrome. Botox should be considered for patients with refractory cases who have received proper counseling about the use of Botox in treating their condition. The clinician should proceed with caution before considering using Botox to treat a patient with pelvic pain, as Botox is only FDA approved for pain related to cervical dystonia. When treating different types of pelvic pain with botulinum toxin A, patients should be closely monitored within the bounds of ethical research standards.

### Urethral Sphincter Dyssynergia/Detrusor Sphincter Dyssynergia

Evidence suggesting decreased postvoid residual, maximum urethral closure pressure, and maximum voiding detrusor pressure after treatment with Botox—while still protecting the upper urinary tract—indicates that patients with detrusor sphincter dyssynergia may benefit from treatment with botulinum toxin A. Gallien et al published one of the largest trials evaluating the effectiveness and safety of Botox in 86 patients, 58 of whom were women, with detrusor sphincter dyssynergia secondary to multiple sclerosis.^54^ Subjects were randomized to intrasphincteric injection of Botox vs placebo. No significant difference in postvoid residual between the 2 groups was found. However, compared to placebo, Botox significantly increased voiding volume (*P*=0.02) and reduced premicturition (*P*=0.02) and maximal detrusor pressures (*P*=0.02). Overall, female-specific data are lacking because many studies included either male-only or combined male/female populations, and additional studies are needed to fully understand the efficacy of botulinum toxin A in treating detrusor sphincter dyssynergia in female patients.

### Colorectal Conditions That May Affect Pelvic Floor Symptoms

#### Hemorrhoids

Treatment of hemorrhoids, especially with traditional hemorrhoidectomy, can cause significant postoperative pain. As postoperative pain for these cases can often be refractory to standard treatment options, development of new methods for pain control is paramount. In 2003, Davies et al reported decreased VAS scores in subjects who received internal anal sphincter injections of botulinum toxin A status post Milligan-Morgan hemorrhoidectomy compared with those who received placebo.^55^ Notably, no difference in narcotic use was seen between groups, and no anal incontinence or other complications were reported for the treatment group. Similarly, a study by Patti et al in 2005 showed improved wound healing after hemorrhoidectomy for third- and fourth-degree piles in patients who received intrasphincteric botulinum toxin injection.^56^ Anorectal manometry was also evaluated preoperatively and postoperatively at intervals, with significant postoperative improvement noted in mean rectal pressure and decreased wound healing time in the Botox treatment group. Anal incontinence was reported for 9 patients, although cases were almost evenly distributed between Botox (n=4) vs placebo (n=5) recipients. A 2008 study by Patti et al assessed the utility of Botox injections for pain relief in the setting of thrombosed external hemorrhoids.^57^ Patients who refused surgical intervention were randomized to receive Botox vs placebo injection. Anorectal manometry was performed before the procedure and 5 days posttreatment. Anal resting pressure, pain, and time to return to work or normal activity were all decreased in the Botox group, while the placebo group had increased analgesic consumption. No patients in either group reported anal incontinence.

#### Anal Fissures

An anal fissure is a tear in the lining of the distal anal canal caudal to the dentate line that most commonly occurs along the posterior midline. The incidence is comparable in both sexes and affects people of varying ages. Clinical symptoms include anal pain during and after defecation, often accompanied by bright red rectal bleeding, and pruritus ani. Acute fissures typically have sharply demarcated fresh mucosal edges and may have granulation tissue along their bases. Chronic fissures typically have rolled edges, characteristically exposing the horizontal fibers of the internal anal sphincter muscle at their bases, and are often accompanied by external skin tags, hypertrophied anal papillae, elevated resting anal pressures, and, rarely, anal stenosis.

Studies assessing botulinum toxin A for treating anal fissures are limited. As early as 1998, Maria et al published a comparison of Botox and saline for the treatment of chronic anal fissures in the *New England Journal of Medicine*.^58^ The Botox group demonstrated higher fissure healing rates (73% vs 13%), with only one subject experiencing transient incontinence of flatus. Botulinum toxin A injection has also been compared with topical glyceryl trinitrate 0.2% for the treatment of patients with chronic anal fissures.^59^ Healing rates were significantly higher (96% vs 60%), and resting anal pressures were decreased (29% vs 14%) in the botulinum toxin A group vs the glyceryl trinitrate group. No side effects were noted in the Botox group, while 20% of the glyceryl trinitrate group complained of headaches. While surgical intervention for anal fissures, particularly with lateral internal sphincterotomy, is considered the therapeutic gold standard, multiple reports have compared lateral internal sphincterotomy to Botox treatment. Although Botox is inferior to lateral internal sphincterotomy when given alone, studies have shown Botox to be less invasive and repeatable and to have a decreased risk of long-term fecal or flatal incontinence compared with lateral internal sphincterotomy.^60,61^ Of note, a study from 2015 showed that treatment with topical diltiazem combined with Botox injection had similar success rates to partial lateral internal sphincterotomy, and the botulinum toxin A + topical diltiazem group had lower rates of fecal incontinence.^62^ In subjects with chronic anal fissures however, the healing rate was significantly higher in the partial lateral internal sphincterotomy group.

## CHALLENGES

Despite demonstrating much utility in treating pelvic floor disorders, especially with regard to evolving therapies, the use of botulinum toxin A is not without challenges. Researchers continue to investigate ideal dosing, injection placement, and administration technique reproducibility, all contributing to the likelihood that recipients will have successful outcomes while minimizing side effects.

**Figure 2.**
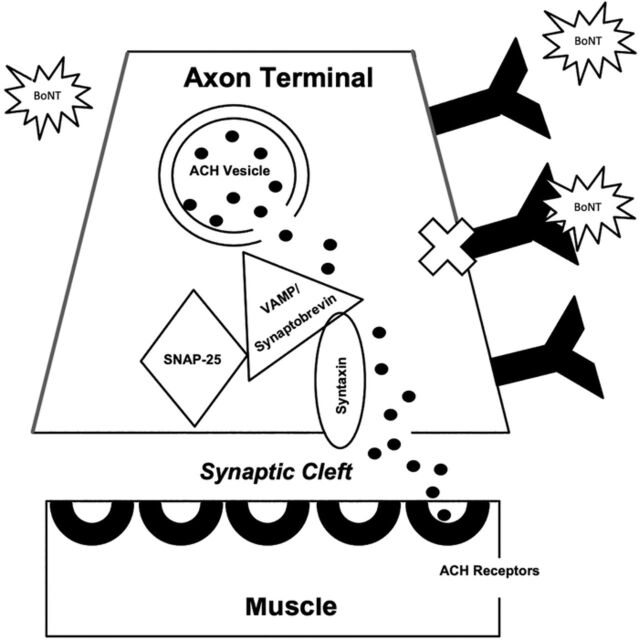
**Mechanism of action of botulinum toxin (BoNT) immunogenicity. After repeated doses of BoNT, antibodies to BoNT may form. When BoNT binds to an antibody, it cannot enter the axon to block formation of the soluble N-ethylmaleimide-sensitive factor attachment protein receptor (SNARE) complex (synaptosome-associated protein of 25kDa [SNAP-25], vesicle-associated membrane protein [VAMP]/synaptobrevin, syntaxin). Therefore, the SNARE complex forms, allowing exocytosis of the neurotransmitter acetylcholine (ACH) from the axon into the synaptic cleft where it can bind end receptors on the muscle, allowing muscle contraction. Thus, efficacy of BoNT is decreased with antibody formation.**

Patients who receive multiple doses can potentially develop immunogenicity to Botox (Figure 2). As Botox is often given repeatedly because of its temporary effect, antibodies may develop, resulting in decreased efficacy over time. The 2 types of antibodies are neutralizing antibodies that bind primarily to the heavy chain of the core neurotoxin and nonneutralizing antibodies that bind to either accessory proteins of the toxin complex or the core neurotoxin in a way that has no effect on biologic effect of the toxin.^63,64^ Although immunogenicity does not signify conclusive formation of neutralizing antibodies, this type of antibody has been specifically associated with a secondary nonresponse or loss of response after a period of successful therapy.^65^ Laboratory tests (mouse diaphragm assay and mouse protection assay) and clinical tests (sternocleidomastoid test, extensor digitorum brevis test, and frowning test) have been developed to detect the presence of Botox antibodies, but not all tests have good sensitivity and specificity.

If ascertainable, treatment of immunogenicity with specific immunoglobulins, immunosuppressants, or plasmapheresis would be ideal, although implementation of each is not without risks and logistic considerations. Prevention of immunogenicity remains the ultimate goal. Minimizing modifiable risk factors (history of shorter dosing intervals, increasing the number of booster doses, and higher doses of Botox per treatment), while acknowledging fixed genetic predisposition, could help reduce the certainty and impact of antigen formation.^66^ Injecting the smallest effective dose of Botox and waiting the longest possible time interval between treatments may decrease the potential for immunogenicity.^67^ Refractory cases may warrant revisiting other standard treatment modalities for the specific pelvic floor condition being treated.

## CONCLUSION

Although botulinum toxin A has well-confirmed efficacy in treating certain urogenital conditions, such as refractory overactive bladder and neurogenic detrusor overactivity, use of Botox to treat different types of pelvic pain, muscle dysfunction, and certain colorectal-related pain issues is promising. Research is needed to provide justification to broaden FDA-approved indications for Botox in treating such conditions and thereby facilitate access to robust, affordable treatment options for patients with these ailments.
